# Supplementary material for: Investigating discharge communication for chronic disease patients in three hospitals in India
Source: PLoS One. 2020 Apr 15;15(4):e0230438. doi: 10.1371/journal.pone.0230438 (PMC7159187; doi:10.1371/journal.pone.0230438)
Supplement: S1 Appendix — (PDF) [file pone.0230438.s001.pdf]

S2 APPENDIX. PREVIOUS HANDOVER RESEARCH STUDY QUESTIONNAIRE USED AS BASIS FOR CURRENT STUDY QUESTIONNAIRE

PATIENT QUESTIONNAIRE

PRIMARY AND SECONDARY CARE INTEGRATION IN LOW AND MIDDLE INCOME COUNTRIES; A CASE STUDY OF CLINICAL HANDOVER IN TWO GOVERNMENT HOSPITALS IN NIGERIA

Instructions: please complete all. **Tick** the appropriate space that suits your best response to each question

All the information below will remain confidential and for the research project use only.

SECTION A: DEMOGRAPHICS

1. Age (years)

   20-29 ……….

   30-39 ……….

   40-49 ……….

   50-59 ……….

   60-69 ……….

   70 and older ……….

   Don't know …………..

2. Sex-
   Male ……….
   Female ……….
3. Religion-
   Christianity ……….
   Islam ……….
   Traditional ……….
   Other ……….

4. Ethnicity-
   Yoruba ……….
   Hausa ……….
   Igbo ……….
   Others ……….

5. Highest level of education -

   Literate but not completed primary school…….

   Primary school completed…..…..

   Secondary School completed …..…..

   University completed…..…..

   Vocational Studies completed…..…..

   None …..…..

6. Occupation-

   Artisan/craftsman …..…..

   Small Business (e.g. carpenter, plumber, petty trading) …..…..

   Average business (e.g. own/rent a shop/shed) …..…..

   Large business (e.g. own a company, employ staff/labour) …..…..

   Junior civil servant (e.g. secretary, assistant) …..…..

   Senior civil servant (e.g. team lead, director)

   Primary/secondary school teacher …..…..

   University lecturer …..…..

   Other (E.g. farmer, street vendor, taxi driver, etc.) …..…..

7. Where do you live?

   Kwara State. …..…..

   FCT …..…..

   Other (please specify) …..….

8. Marital status-

   Single …..…..

   Married …..…..

   Divorced …..…..

   Widow(er) …..…..

SECTION B: PAST MEDICAL HISTORY

9. What medical condition has brought you to hospital today?

   Diabetes …..…..

   Hypertension …..…..

   Heart disease …..…..

   Don't' know …..…..

   Other (Please explain) ………………………………………………………………………………

10. Have you visited any other health centre/doctor/nurse/chemist before coming here?

    Yes …..…..

    No …..…..

11. If your answer to the last question 10 is yes, please specify- (You can choose more than one)

Local Government Health Centre …..…..

Another general hospital …..…..

Private hospital …..…..

Private Doctor or nurse …..…..

Traditional healer …..…..

Religious healer …..…..Other

12. For how long have you used this other health service before coming to this hospital (or at the same time as occasional visits to this hospital)?

Less than 6 months …..…..

6months – 1year …..…..

1year – 2years …..…..

More than 2 years …..…..

Don't know …..…..

13. For how long have you used this current hospital?

Less than 6 months …..…..

6months – 1year …..…..

1year – 2years …..…..

More than 2 years …..…..

Don't know …..…..

SECTION C: PREVIOUS CARE HISTORY

14. What has made you visit this hospital today instead of the health service you mentioned in question 11?

Cost is lower here …..…..

This hospital is nearer to where I live …..…..

The other clinic or someone else referred (sent) me here …..…..

Employment reasons …..…..

Quality of care is better here …..…..

Personal reasons …..…..

Other …..…..

15. Do you have any document, notes or other pieces of paper from your previous health provider to this hospital?

Yes …..…..

No …..….. **[if your answer is 'No' please proceed to question 17]**

16. Do you have that note with you today?

Yes …..…..

No …..…..

17. If your answer to question 16 is No, please provide a reason-
    Forgot it at home ……….
    Lost it ……….
    I've always had it but never used it ……….
    I've never been asked for it here ……….
    My children/spouse handle such documents, so I don't know ……….

18. If your answer to question 15 was yes, please how did you get it- (please may I see it?)
    I was given one without asking ……….
    I had to ask for a document ……….
    In this hospital I need one before I am can be attended to ……….

19. Note for researcher: is the referral paper any of the following?
    Picture card ……..
    Letter ……..
    Referral form ……..

20. Does it contain any of the following:

    Past medical history ……..

    Patient's symptoms ……..

    Tests already performed ……..

    Tests to be performed ……..

    Differential diagnosis ……..Medication ……..

<u>Thank you for your participation</u>

<u>PATIENT QUESTIONNAIRE (PART II)</u>

TITLE OF RESEARCH PROJECT: PRIMARY AND SECONDARY CARE INTEGRATION IN LOW AND MIDDLE INCOME COUNTRIES; A CASE STUDY OF CLINICAL HANDOVER IN TWO GOVERNMENT HOSPITALS IN NIGERIA

Instructions: please answer all. Tick the appropriate space that suits your best response to each question. You may choose more than one option where possible

1. Do you plan to return to your previous care provider (nurse, doctor, etc) you visited before coming here if you had one?

Yes ……..

No ……..

Don't know ……..

2. If yes, how will you explain to them what was done for you here?

   I told the doctor here to explain to my child/spouse ……..

   I asked the doctor here to explain to me so I can tell the other doctors I may see ……..

   The doctor here gave me a note to take back to my other health provider ……..

   Don't know ……..

3. In your opinion does having a note make the care you received here better than other places you have visited?

   Yes ……..

   No ……..

   Don't know ……..

4. If yes, why?

   Because I don't know how to explain my condition and the note helps ……..

   It helps me to get attended to faster ……..

   I feel it's more professional ……..

   I don't know ……..

5. If no, why?

   I don't know ……..

   Everyone receives the same standard of care regardless ……..

   It's a waste of time ……..

   Other (Please explain briefly) ……………………………………………………………………………………………………… ……………………………………………………………………………

6. Did the doctor explain your condition to you?

   Yes ……..

   No ……..

   Don't know ……..

7. What were you asked to do after leaving here?

   Come back ……..

   Go to local hospital ……..

   Get some new medication ……..

   Continue with old medication ……..

   Do some tests ……..

8. Did the doctor give you a **note to give your other doctor** or a **note to come back here**?

   Yes ……..

   No ……..

Don't know ……..

9. If yes please may I see it?
10. Note for researcher- is the referral paper any of the following?

Patient card/note ……..

Letter ……..

Referral form ……..

11. Does it contain any of the following:

Past medical history ……..

Patient's symptoms ……..

Tests already performed ……..

Tests to be performed ……..

Differential diagnosis ……..

Medication ……..

<u>Thank you for your participation</u>
