# Supplementary material for: Investigating discharge communication for chronic disease patients in three hospitals in India
Source: PLoS One. 2020 Apr 15;15(4):e0230438. doi: 10.1371/journal.pone.0230438 (PMC7159187; doi:10.1371/journal.pone.0230438)
Supplement: S2 Appendix — (PDF) [file pone.0230438.s002.pdf]

S2 APPENDIX. Baseline characteristics and adverse health outcomes

Table 1. Participant demographic and adverse health outcome information

Characteristic	Hospital 1 (n=308) Frequency (%)	Hospital 2 (n=64) Frequency (%)	Hospital 3 (n=174) Frequency (%)
**Sex**			
Male	190 (61.7)	12 (18.8)	103 (59.2)
Female	118 (38.3)	52 (81.3)	71 (40.8)
**Age Group (Years)**			
18–49	47 (15.3)	17 (26.6)	34 (19.5)
50–69	167 (54.2)	40 (62.5)	89 (51.1)
≥70	94 (30.5)	7 (10.9)	51 (29.3)
**Level of Education**			
Illiterate	37 (12.0)	4 (6.3)	50 (28.7)
Literate with partial or completed primary school education	167 (54.2)	34 (53.1)	57 (32.8)
Complete secondary school education	75 (24.4)	16 (25.0)	41 (23.6)
Complete higher school/vocational studies	28 (9.1)	10 (15.6)	14 (8.0)
University graduate or above	1 (0.3)	0 (0)	12 (6.9)
**Employment Status**			
Employed	86 (27.9)	17 (26.6)	61 (35.1)
Unemployed	220 (71.4)	47 (73.4)	102 (58.6)
Retired	0 (0)	0 (0)	11 (6.3)
No data*	2 (0.6)	0 (0)	0 (0)
**Time Taken to Reach Hospital**			
>1 hour	152 (49.4)	32 (50.0)	127 (73.0)
1-4 hours	152 (49.4)	32 (50.0)	46 (26.4)
>4 hours	3 (1.0)	0 (0)	1 (0.6)
No data*	1 (0.3)	0 (0)	0 (0)
**Chronic NCDs†**			
Diabetes	101 (32.8)	26 (40.6)	30 (17.2)
Cardiovascular Disease	154 (50)	17 (26.6)	47 (27.0)
Chronic Respiratory Disease	135 (43.8)	18 (28.1)	94 (54.0)
Hypertension	103 (33.4)	24 (37.5)	44 (25.3)
**Number of Chronic NCDs (per patient)**			
1	181 (58.8)	47 (73.4)	137 (78.7)
2	81 (26.3)	14 (21.9)	33 (19.0)
3	34 (11.0)	2 (3.1)	4 (2.3)
4	12 (3.9)	1 (1.6)	0 (0)
**Adverse Health Outcomes at 5-week follow-up**			
Death	1 (0.3)	0 (0)	18 (10.3)
Hospital Readmission	21 (6.8)	1 (1.6)	11 (6.3)
Self-reported deterioration of NCD/s	32 (10.4)	1 (1.6)	6 (3.4)
**Adverse Health Outcomes at 18-week follow-up**			
Death	6 (1.9)	0 (0)	19 (10.9)
Hospital Readmission	46 (14.9)	6 (9.4)	17 (9.8)
Self-reported deterioration of NCD/s	51 (16.6)	2 (3.1)	9 (5.2)

* No data = missing responses
† Please note that participants could select more than one answer for this question
