# Supplementary material for: Investigating discharge communication for chronic disease patients in three hospitals in India
Source: PLoS One. 2020 Apr 15;15(4):e0230438. doi: 10.1371/journal.pone.0230438 (PMC7159187; doi:10.1371/journal.pone.0230438)
Supplement: S3 Appendix — (PDF) [file pone.0230438.s003.pdf]

S3 APPENDIX. EXAMPLE OF A STRUCTURED DISCHARGE DOCUMENT PROVIDED TO PATIENTS

Figure 1. Picture of a structured “discharge slip” from a study hospital

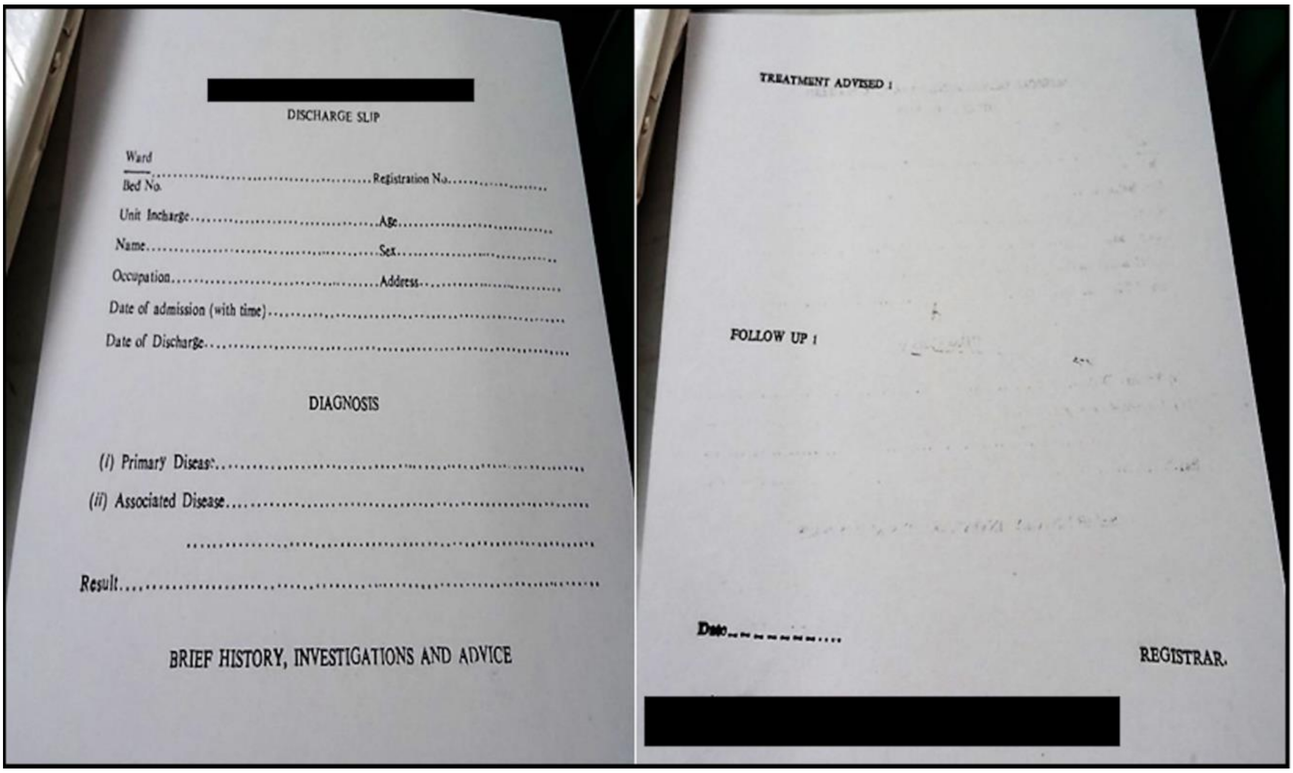

[redacted]

DISCHARGE SLIP

Ward
Bed No. .... Registration No. ....

Unit Incharge........................ Age........................

Name........................ Sex........................

Occupation........................ Address........................

Date of admission (with time)........................

Date of Discharge........................

DIAGNOSIS

(i) Primary Disease........................

(ii) Associated Disease........................

....

Result........................

BRIEF HISTORY, INVESTIGATIONS AND ADVICE

TREATMENT ADVISED :

FOLLOW UP :

Date..........

REGISTRAR.

[redacted]
