# Supplementary material for: Investigating discharge communication for chronic disease patients in three hospitals in India
Source: PLoS One. 2020 Apr 15;15(4):e0230438. doi: 10.1371/journal.pone.0230438 (PMC7159187; doi:10.1371/journal.pone.0230438)

S4 APPENDIX. SUMMARY OF PARTICIPANT INCLUSION AND EXCLUSION THROUGHOUT STUDY

Figure 2. Flowchart illustrating the inclusion and exclusion of participants throughout the study

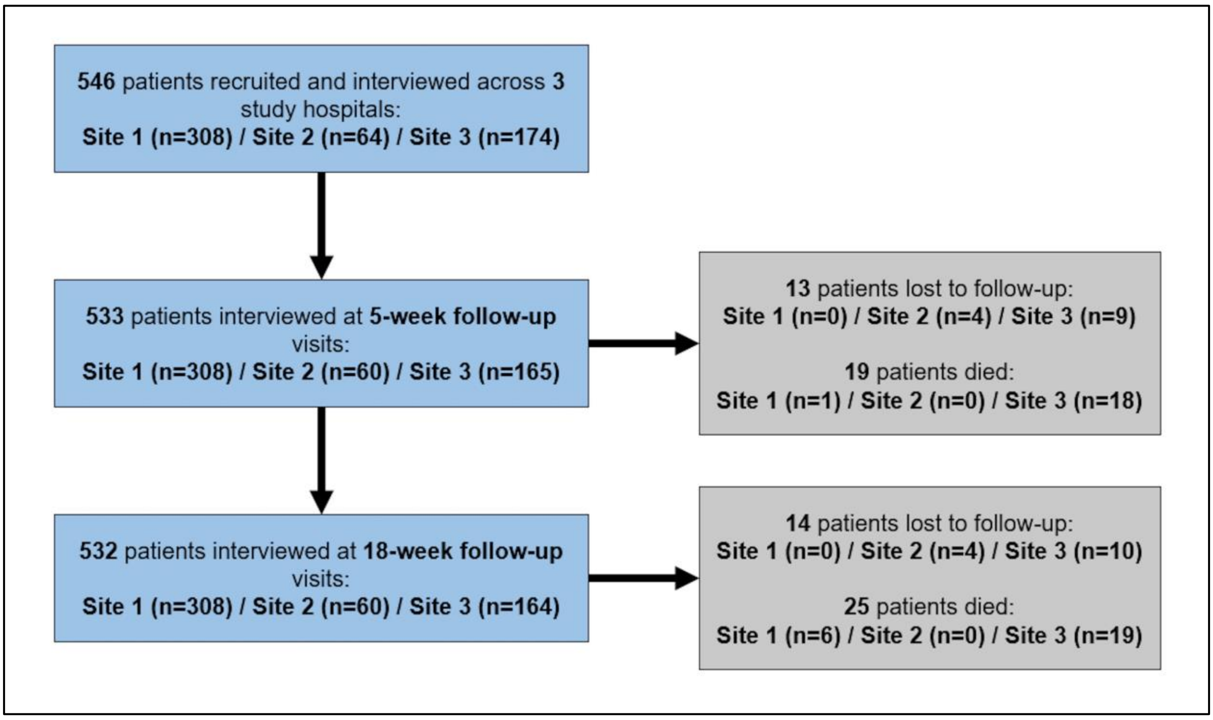

Supplement: S4 Appendix — (PDF) [file pone.0230438.s004.pdf]
