# Supplementary material for: Investigating discharge communication for chronic disease patients in three hospitals in India
Source: PLoS One. 2020 Apr 15;15(4):e0230438. doi: 10.1371/journal.pone.0230438 (PMC7159187; doi:10.1371/journal.pone.0230438)
Supplement: S5 Appendix — (PDF) [file pone.0230438.s005.pdf]

S5 APPENDIX. RESULTS OF UNADJUSTED REGRESSION ANALYSES

Table 2. Unadjusted associations between receiving low-quality discharge information and the likelihood of experiencing adverse health outcomes within five weeks and eighteen of discharge

**Death within 5 weeks of discharge**	**Unadjusted odds ratios**			**Death within 18 weeks of discharge**	**Unadjusted odds ratios**		
	OR	95% CI	p-value		OR	95% CI	p-value
No. of items of key documents discharge information[*]				*No. of items of key documented discharge information*[*]			
0 to 2 items	4.26	1.64-11.05	0.003[§]	0 to 2 items	3.58	1.58-8.13	0.002[†]
No. of items of key verbal discharge information[§]				*No. of items of key verbal discharge information*[§]			
0 to 2 items	13.79	0.83-229.90	0.068	0 to 2 items	3.32	0.89-12.40	0.075
**Hospital readmission within 5 weeks of discharge**				**Hospital readmission within 18 weeks of discharge**			
No. of items of key documented discharge information[*]				*No. of items of key documented discharge information*[*]			
0 to 2 items	0.69	0.31-1.55	0.374	0 to 2 items	0.86	0.49-1.49	0.580
No. of items of key verbal discharge information[§]				*No. of items of key verbal discharge information*[§]			
0 to 2 items	0.78	0.37-1.66	0.518	0 to 2 items	0.77	0.45-1.34	0.364
**Self-reported deterioration of NCD/s within 5 weeks of discharge**				**Self-reported deterioration of NCD/s within 18 weeks of discharge**			
No. of items of key documented discharge information[*]				*No. of items of key documented discharge information*[*]			
0 to 2 items	1.33	0.68-2.58	0.406	0 to 2 items	1.50	0.87-2.58	0.145
No. of items of key verbal discharge information[§]				*No. of items of key verbal discharge information*[§]			
0 to 2 items	0.53	0.27-1.03	0.063	0 to 2 items	0.40	0.23-0.68	0.001[†]

***** Odds ratios represent association with receipt of 0 to 2 items of key documented information on discharge notes

† Statistically significant at $p<0.05$

§ Odds ratios represent association with receipt of 0 to 2 items of key verbal information during discharge consultation
