# Supplementary material for: Investigating discharge communication for chronic disease patients in three hospitals in India
Source: PLoS One. 2020 Apr 15;15(4):e0230438. doi: 10.1371/journal.pone.0230438 (PMC7159187; doi:10.1371/journal.pone.0230438)
Supplement: S6 Appendix — (PDF) [file pone.0230438.s006.pdf]

S6 APPENDIX. SUMMARY OF GOODNESS OF FIT TEST RESULTS FOR ALL ADJUSTED MULTIVARIATE ANALYSES

Table 3. Results of goodness-of-fit tests for the adjusted multivariate analyses

**Models with adverse health outcomes within 5 weeks of discharge***	p-value	**Models with adverse health outcomes within 18 weeks of discharge***	p-value
Death	<0.001	Death	0.001
Hospital readmission	0.646	Hospital readmission	0.658
Self-reported deterioration of NCD/s	0.128	Self-reported deterioration of NCD/s	0.009

*Adjusted for the following independent variables: sex, age group (18-49/50-69/70yrs+), education level (up to primary school-level/secondary school-level/higher school-level or more), employment status (unemployed/employed/retired), usual time taken to reach hospital (<1 hour/1-4 hours/>4 hours), number of chronic NCDs (1/2/3/4) and hospital site (1/2/3)
