# Supplementary material for: Investigating discharge communication for chronic disease patients in three hospitals in India
Source: PLoS One. 2020 Apr 15;15(4):e0230438. doi: 10.1371/journal.pone.0230438 (PMC7159187; doi:10.1371/journal.pone.0230438)
Supplement: S7 Appendix — (PDF) [file pone.0230438.s007.pdf]

S7 APPENDIX. SENSITIVITY ANALYSIS

During analysis, it became apparent that the number of hospital readmissions recorded in our patient population was likely to be incomplete. This is because no data was collected about the period after initial discharge until death for deceased patients. Anecdotal reports from Indian clinicians suggest that for chronic NCD patients, exacerbations before death would usually result in an admission - even in the case of a terminal patient. That given, our results appear to represent a lower bound on the proportion of patients readmitted. We therefore ran further regression analyses based on the assumption that all patients who died had also been readmitted. No significant associations were found and all adjusted point estimates regarding associations with low-quality verbal discharge information leaned in the direction of a decreased likelihood of hospital readmission within five and eighteen weeks follow-up. Conversely, all adjusted point estimates regarding associations with low-quality discharge notes leaned in the direction of an increased likelihood of hospital readmission within five and eighteen weeks follow-up. Such results define an upper bound on the proportion of patients readmitted.

Table 4. Results of sensitivity analyses displaying associations between receiving low-quality discharge information and the likelihood of experiencing death or hospital readmission within five and eighteen weeks of discharge

**Readmission or death within 5 weeks of discharge**	**Unadjusted odds ratios**			**Adjusted odds ratios***		
	OR	95% CI	p-value	OR	95% CI	p-value
No. of items of key documented discharge information†						
0 to 2 items	1.41	0.79-2.53	0.247	1.22	0.65-2.29	0.530
No. of items of key verbal discharge information§						
0 to 2 items	1.41	0.70-2.85	0.340	0.88	0.38-2.02	0.756
**Readmission or death within 18 weeks of discharge**						
No. of items of key documented discharge information†						
0 to 2 items	1.31	0.82-2.07	0.254	1.13	0.69-1.83	0.629
No. of items of key verbal discharge information§						
0 to 2 items	1.09	0.65-1.84	0.741	0.94	0.53-1.68	0.835

*Adjusted for the following independent variables: sex, age group (18-49/50-69/70yrs+), education level (up to primary school-level/secondary school-level/higher school-level or more), employment status (unemployed/employed/retired), usual time taken to reach hospital (<1 hour/1-4 hours/>4 hours), number of chronic NCDs (1/2/3/4) and hospital site (1/2/3)
† Odds ratios represent association with receipt of 0 to 2 items of key documented information on discharge notes
§ Odds ratios represent association with receipt of 0 to 2 items of key verbal information during discharge consultation

Table 5. Results of goodness-of-fit tests for the adjusted sensitivity analyses

**Model with adverse health outcomes within 5 weeks of discharge***	p-value	**Model with adverse health outcomes within 18 weeks of discharge***	p-value
Death or Readmission	0.008	Death or Readmission	0.190

*Adjusted for the following independent variables: sex, age group (18-49/50-69/70yrs+), education level (up to primary school-level/secondary school-level/higher school-level or more), employment status (unemployed/employed/retired), usual time taken to reach hospital (<1 hour/1-4 hours/>4 hours), number of chronic NCDs (1/2/3/4) and hospital site (1/2/3).
